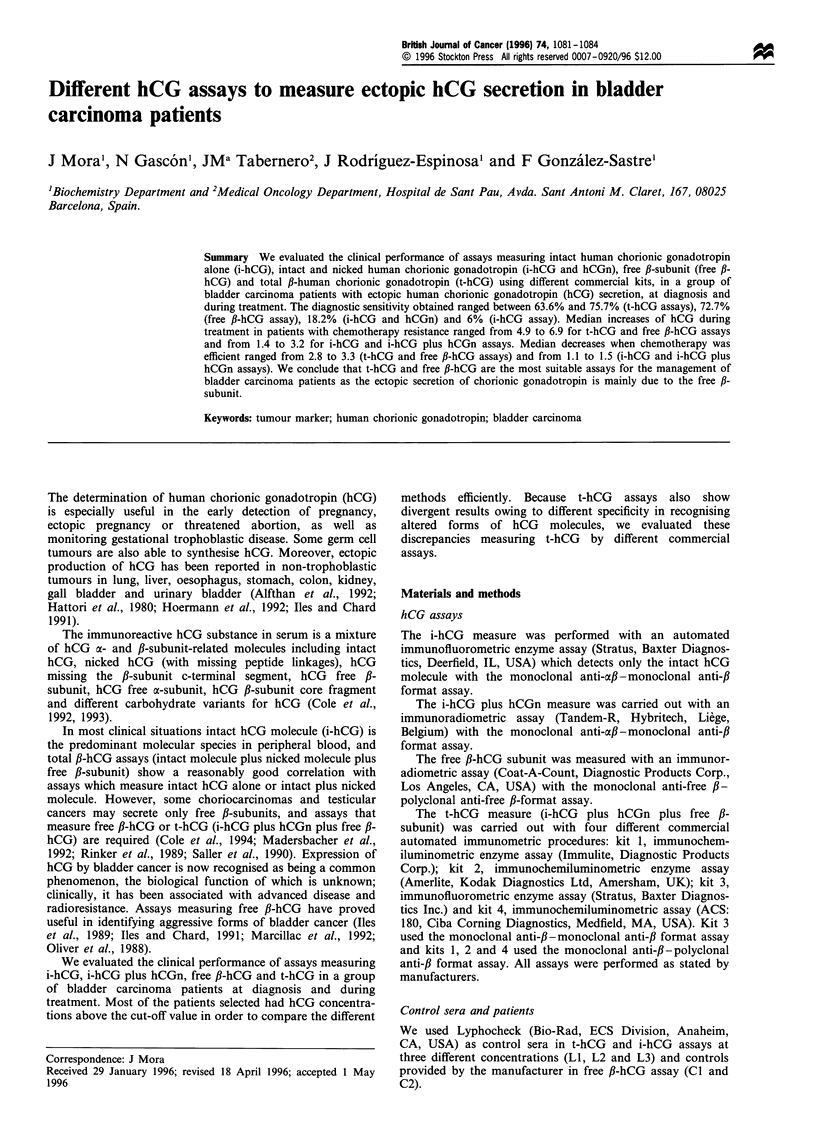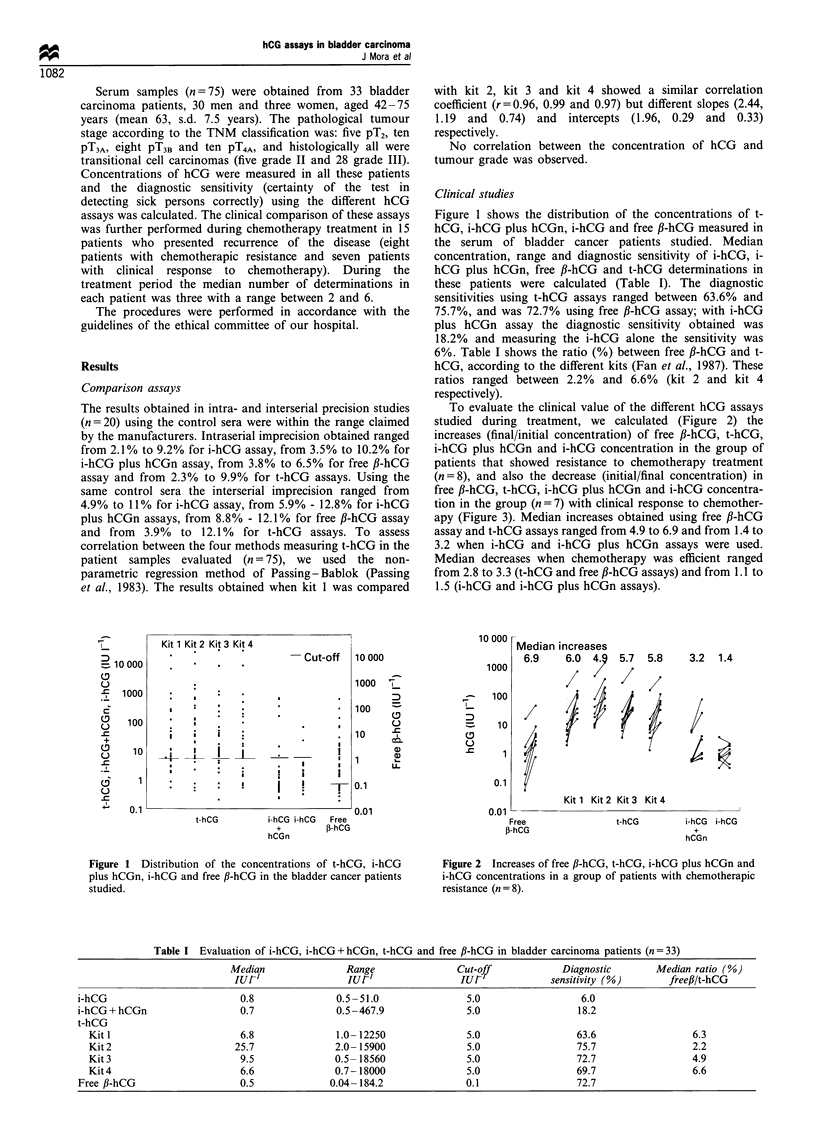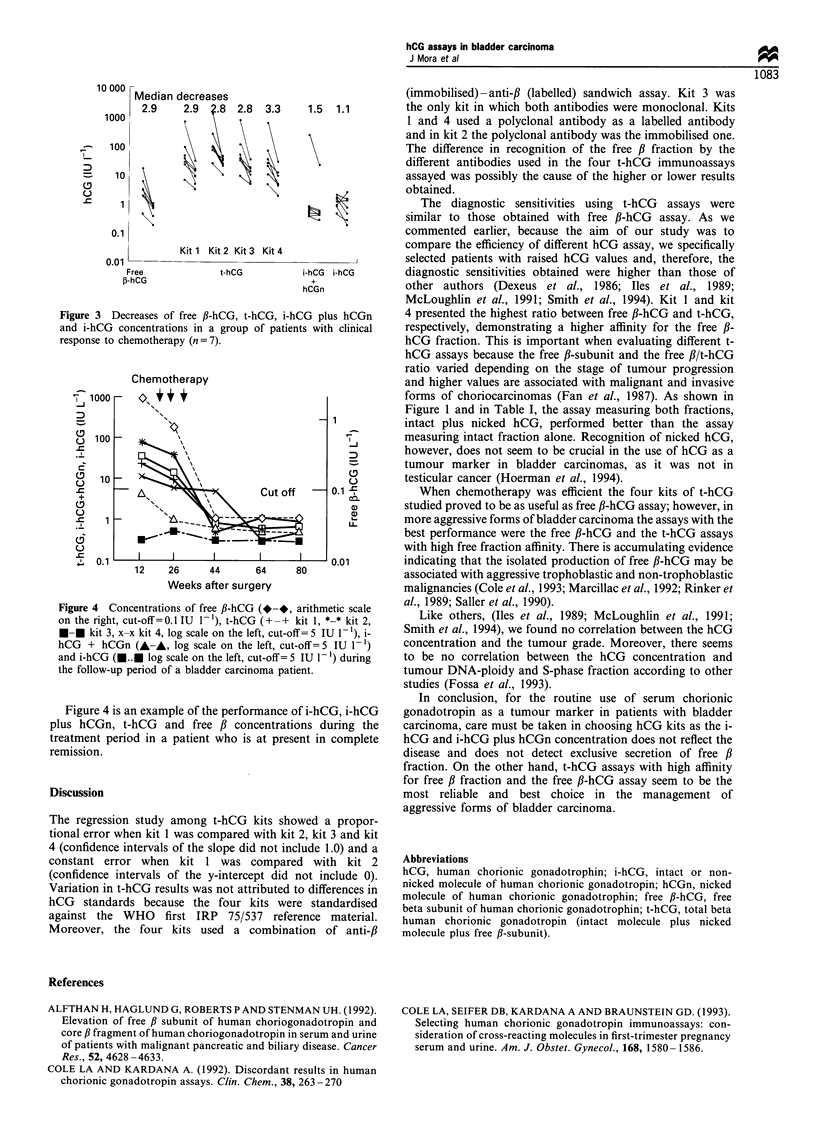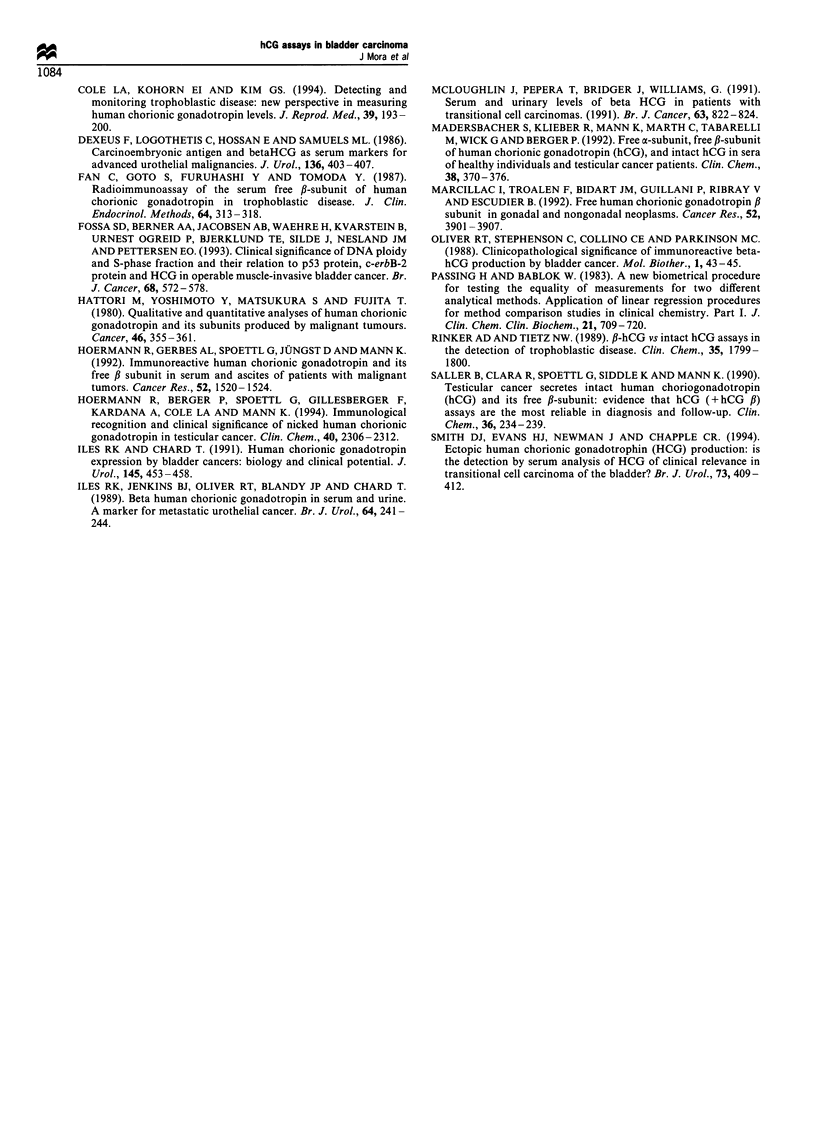# Different hCG assays to measure ectopic hCG secretion in bladder carcinoma patients.

**DOI:** 10.1038/bjc.1996.493

**Published:** 1996-10

**Authors:** J. Mora, N. Gascón, J. M. Tabernero, J. Rodríguez-Espinosa, F. González-Sastre

**Affiliations:** Biochemistry Department, Hospital de Sant Pau, Barcelona, Spain.

## Abstract

We evaluated the clinical performance of assays measuring intact human chorionic gonadotropin alone (i-hCG), intact and nicked human chorionic gonadotropin (i-hCG and hCGn), free beta-subunit (free beta-hCG) and total beta-human chorionic gonadotropin (t-hCG) using different commercial kits, in a group of bladder carcinoma patients with ectopic human chorionic gonadotropin (hCG) secretion, at diagnosis and during treatment. The diagnostic sensitivity obtained ranged between 63.6% and 75.7% (t-hCG assays), 72.7% (free beta-hCG assay), 18.2% (i-hCG and hCGn) and 6% (i-hCG assay). Median increases of hCG during treatment in patients with chemotherapy resistance ranged from 4.9 to 6.9 for t-hCG and free beta-hCG assays and from 1.4 to 3.2 for i-hCG and i-hCG plus hCGn assays. Median decreases when chemotherapy was efficient ranged from 2.8 to 3.3 (t-hCG and free beta-hCG assays) and from 1.1 to 1.5 (i-hCG and i-hCG plus hCGn assays). We conclude that t-hCG and free beta-hCG are the most suitable assays for the management of bladder carcinoma patients as the ectopic secretion of chorionic gonadotropin is mainly due to the free beta-subunit.


British Journal of Cancer (1996) 74, 1081-1084
© 1996 Stockton Press All rights reserved 0007-0920/96 $12.00

### Different hCG assays to measure ectopic hCG secretion in bladder carcinoma patients

J Mora[1], N Gascón[1], JMª Tabernero[2], J Rodríguez-Espinosa[1] and F González-Sastre[1]

[1]*Biochemistry Department and* [2]*Medical Oncology Department, Hospital de Sant Pau, Avda. Sant Antoni M. Claret, 167, 08025 Barcelona, Spain.*

**Summary** We evaluated the clinical performance of assays measuring intact human chorionic gonadotropin alone (i-hCG), intact and nicked human chorionic gonadotropin (i-hCG and hCGn), free β-subunit (free β-hCG) and total β-human chorionic gonadotropin (t-hCG) using different commercial kits, in a group of bladder carcinoma patients with ectopic human chorionic gonadotropin (hCG) secretion, at diagnosis and during treatment. The diagnostic sensitivity obtained ranged between 63.6% and 75.7% (t-hCG assays), 72.7% (free β-hCG assay), 18.2% (i-hCG and hCGn) and 6% (i-hCG assay). Median increases of hCG during treatment in patients with chemotherapy resistance ranged from 4.9 to 6.9 for t-hCG and free β-hCG assays and from 1.4 to 3.2 for i-hCG and i-hCG plus hCGn assays. Median decreases when chemotherapy was efficient ranged from 2.8 to 3.3 (t-hCG and free β-hCG assays) and from 1.1 to 1.5 (i-hCG and i-hCG plus hCGn assays). We conclude that t-hCG and free β-hCG are the most suitable assays for the management of bladder carcinoma patients as the ectopic secretion of chorionic gonadotropin is mainly due to the free β-subunit.

**Keywords:** tumour marker; human chorionic gonadotropin; bladder carcinoma

The determination of human chorionic gonadotropin (hCG) is especially useful in the early detection of pregnancy, ectopic pregnancy or threatened abortion, as well as monitoring gestational trophoblastic disease. Some germ cell tumours are also able to synthesise hCG. Moreover, ectopic production of hCG has been reported in non-trophoblastic tumours in lung, liver, oesophagus, stomach, colon, kidney, gall bladder and urinary bladder (Alfthan *et al.*, 1992; Hattori *et al.*, 1980; Hoermann *et al.*, 1992; Iles and Chard 1991).

The immunoreactive hCG substance in serum is a mixture of hCG α- and β-subunit-related molecules including intact hCG, nicked hCG (with missing peptide linkages), hCG missing the β-subunit c-terminal segment, hCG free β-subunit, hCG free α-subunit, hCG β-subunit core fragment and different carbohydrate variants for hCG (Cole *et al.*, 1992, 1993).

In most clinical situations intact hCG molecule (i-hCG) is the predominant molecular species in peripheral blood, and total β-hCG assays (intact molecule plus nicked molecule plus free β-subunit) show a reasonably good correlation with assays which measure intact hCG alone or intact plus nicked molecule. However, some choriocarcinomas and testicular cancers may secrete only free β-subunits, and assays that measure free β-hCG or t-hCG (i-hCG plus hCGn plus free β-hCG) are required (Cole *et al.*, 1994; Madersbacher *et al.*, 1992; Rinker *et al.*, 1989; Saller *et al.*, 1990). Expression of hCG by bladder cancer is now recognised as being a common phenomenon, the biological function of which is unknown; clinically, it has been associated with advanced disease and radioresistance. Assays measuring free β-hCG have proved useful in identifying aggressive forms of bladder cancer (Iles *et al.*, 1989; Iles and Chard, 1991; Marcillac *et al.*, 1992; Oliver *et al.*, 1988).

We evaluated the clinical performance of assays measuring i-hCG, i-hCG plus hCGn, free β-hCG and t-hCG in a group of bladder carcinoma patients at diagnosis and during treatment. Most of the patients selected had hCG concentrations above the cut-off value in order to compare the different methods efficiently. Because t-hCG assays also show divergent results owing to different specificity in recognising altered forms of hCG molecules, we evaluated these discrepancies measuring t-hCG by different commercial assays.

Correspondence: J Mora
Received 29 January 1996; revised 18 April 1996; accepted 1 May 1996

### Materials and methods

### *hCG assays*

The i-hCG measure was performed with an automated immunofluorometric enzyme assay (Stratus, Baxter Diagnostics, Deerfield, IL, USA) which detects only the intact hCG molecule with the monoclonal anti-αβ–monoclonal anti-β format assay.

The i-hCG plus hCGn measure was carried out with an immunoradiometric assay (Tandem-R, Hybritech, Liège, Belgium) with the monoclonal anti-αβ–monoclonal anti-β format assay.

The free β-hCG subunit was measured with an immunoradiometric assay (Coat-A-Count, Diagnostic Products Corp., Los Angeles, CA, USA) with the monoclonal anti-free β–polyclonal anti-free β-format assay.

The t-hCG measure (i-hCG plus hCGn plus free β-subunit) was carried out with four different commercial automated immunometric procedures: kit 1, immunochemiluminometric enzyme assay (Immulite, Diagnostic Products Corp.); kit 2, immunochemiluminometric enzyme assay (Amerlite, Kodak Diagnostics Ltd, Amersham, UK); kit 3, immunofluorometric enzyme assay (Stratus, Baxter Diagnostics Inc.) and kit 4, immunochemiluminometric assay (ACS: 180, Ciba Corning Diagnostics, Medfield, MA, USA). Kit 3 used the monoclonal anti-β–monoclonal anti-β format assay and kits 1, 2 and 4 used the monoclonal anti-β–polyclonal anti-β format assay. All assays were performed as stated by manufacturers.

### *Control sera and patients*

We used Lyphocheck (Bio-Rad, ECS Division, Anaheim, CA, USA) as control sera in t-hCG and i-hCG assays at three different concentrations (L1, L2 and L3) and controls provided by the manufacturer in free β-hCG assay (C1 and C2).

Serum samples ($n=75$) were obtained from 33 bladder carcinoma patients, 30 men and three women, aged 42–75 years (mean 63, s.d. 7.5 years). The pathological tumour stage according to the TNM classification was: five $pT_2$, ten $pT_{3A}$, eight $pT_{3B}$ and ten $pT_{4A}$, and histologically all were transitional cell carcinomas (five grade II and 28 grade III). Concentrations of hCG were measured in all these patients and the diagnostic sensitivity (certainty of the test in detecting sick persons correctly) using the different hCG assays was calculated. The clinical comparison of these assays was further performed during chemotherapy treatment in 15 patients who presented recurrence of the disease (eight patients with chemotherapic resistance and seven patients with clinical response to chemotherapy). During the treatment period the median number of determinations in each patient was three with a range between 2 and 6.

The procedures were performed in accordance with the guidelines of the ethical committee of our hospital.

### Results

*Comparison assays*

The results obtained in intra- and interserial precision studies ($n=20$) using the control sera were within the range claimed by the manufacturers. Intraserial imprecision obtained ranged from 2.1% to 9.2% for i-hCG assay, from 3.5% to 10.2% for i-hCG plus hCGn assay, from 3.8% to 6.5% for free $\beta$-hCG assay and from 2.3% to 9.9% for t-hCG assays. Using the same control sera the interserial imprecision ranged from 4.9% to 11% for i-hCG assay, from 5.9% - 12.8% for i-hCG plus hCGn assays, from 8.8% - 12.1% for free $\beta$-hCG assay and from 3.9% to 12.1% for t-hCG assays. To assess correlation between the four methods measuring t-hCG in the patient samples evaluated ($n=75$), we used the non-parametric regression method of Passing–Bablok (Passing *et al.*, 1983). The results obtained when kit 1 was compared with kit 2, kit 3 and kit 4 showed a similar correlation coefficient ($r=0.96$, 0.99 and 0.97) but different slopes (2.44, 1.19 and 0.74) and intercepts (1.96, 0.29 and 0.33) respectively.

No correlation between the concentration of hCG and tumour grade was observed.

*Clinical studies*

Figure 1 shows the distribution of the concentrations of t-hCG, i-hCG plus hCGn, i-hCG and free $\beta$-hCG measured in the serum of bladder cancer patients studied. Median concentration, range and diagnostic sensitivity of i-hCG, i-hCG plus hCGn, free $\beta$-hCG and t-hCG determinations in these patients were calculated (Table I). The diagnostic sensitivities using t-hCG assays ranged between 63.6% and 75.7%, and was 72.7% using free $\beta$-hCG assay; with i-hCG plus hCGn assay the diagnostic sensitivity obtained was 18.2% and measuring the i-hCG alone the sensitivity was 6%. Table I shows the ratio (%) between free $\beta$-hCG and t-hCG, according to the different kits (Fan *et al.*, 1987). These ratios ranged between 2.2% and 6.6% (kit 2 and kit 4 respectively).

To evaluate the clinical value of the different hCG assays studied during treatment, we calculated (Figure 2) the increases (final/initial concentration) of free $\beta$-hCG, t-hCG, i-hCG plus hCGn and i-hCG concentration in the group of patients that showed resistance to chemotherapy treatment ($n=8$), and also the decrease (initial/final concentration) in free $\beta$-hCG, t-hCG, i-hCG plus hCGn and i-hCG concentration in the group ($n=7$) with clinical response to chemotherapy (Figure 3). Median increases obtained using free $\beta$-hCG assay and t-hCG assays ranged from 4.9 to 6.9 and from 1.4 to 3.2 when i-hCG and i-hCG plus hCGn assays were used. Median decreases when chemotherapy was efficient ranged from 2.8 to 3.3 (t-hCG and free $\beta$-hCG assays) and from 1.1 to 1.5 (i-hCG and i-hCG plus hCGn assays).

**Figure 1** Distribution of the concentrations of t-hCG, i-hCG plus hCGn, i-hCG and free $\beta$-hCG in the bladder cancer patients studied.

**Figure 2** Increases of free $\beta$-hCG, t-hCG, i-hCG plus hCGn and i-hCG concentrations in a group of patients with chemotherapic resistance ($n=8$).

**Table I** Evaluation of i-hCG, i-hCG+hCGn, t-hCG and free $\beta$-hCG in bladder carcinoma patients ($n=33$)

| | *Median* $IU\,l^{-1}$ | *Range* $IU\,l^{-1}$ | *Cut-off* $IU\,l^{-1}$ | *Diagnostic sensitivity (%)* | *Median ratio (%) free$\beta$/t-hCG* |
|---|---|---|---|---|---|
| i-hCG | 0.8 | 0.5–51.0 | 5.0 | 6.0 | |
| i-hCG + hCGn | 0.7 | 0.5–467.9 | 5.0 | 18.2 | |
| t-hCG | | | | | |
| Kit 1 | 6.8 | 1.0–12250 | 5.0 | 63.6 | 6.3 |
| Kit 2 | 25.7 | 2.0–15900 | 5.0 | 75.7 | 2.2 |
| Kit 3 | 9.5 | 0.5–18560 | 5.0 | 72.7 | 4.9 |
| Kit 4 | 6.6 | 0.7–18000 | 5.0 | 69.7 | 6.6 |
| Free $\beta$-hCG | 0.5 | 0.04–184.2 | 0.1 | 72.7 | |

**Figure 3** Decreases of free $\beta$-hCG, t-hCG, i-hCG plus hCGn and i-hCG concentrations in a group of patients with clinical response to chemotherapy ($n=7$).

**Figure 4** Concentrations of free $\beta$-hCG (◆–◆, arithmetic scale on the right, cut-off = 0.1 IU $l^{-1}$), t-hCG (+–+ kit 1, *-* kit 2, ■-■ kit 3, x–x kit 4, log scale on the left, cut-off = 5 IU $l^{-1}$), i-hCG + hCGn (▲-▲, log scale on the left, cut-off = 5 IU $l^{-1}$) and i-hCG (■..■ log scale on the left, cut-off = 5 IU $l^{-1}$) during the follow-up period of a bladder carcinoma patient.

Figure 4 is an example of the performance of i-hCG, i-hCG plus hCGn, t-hCG and free $\beta$ concentrations during the treatment period in a patient who is at present in complete remission.

### Discussion

The regression study among t-hCG kits showed a proportional error when kit 1 was compared with kit 2, kit 3 and kit 4 (confidence intervals of the slope did not include 1.0) and a constant error when kit 1 was compared with kit 2 (confidence intervals of the y-intercept did not include 0). Variation in t-hCG results was not attributed to differences in hCG standards because the four kits were standardised against the WHO first IRP 75/537 reference material. Moreover, the four kits used a combination of anti-$\beta$ (immobilised)–anti-$\beta$ (labelled) sandwich assay. Kit 3 was the only kit in which both antibodies were monoclonal. Kits 1 and 4 used a polyclonal antibody as a labelled antibody and in kit 2 the polyclonal antibody was the immobilised one. The difference in recognition of the free $\beta$ fraction by the different antibodies used in the four t-hCG immunoassays assayed was possibly the cause of the higher or lower results obtained.

The diagnostic sensitivities using t-hCG assays were similar to those obtained with free $\beta$-hCG assay. As we commented earlier, because the aim of our study was to compare the efficiency of different hCG assay, we specifically selected patients with raised hCG values and, therefore, the diagnostic sensitivities obtained were higher than those of other authors (Dexeus *et al.*, 1986; Iles *et al.*, 1989; McLoughlin *et al.*, 1991; Smith *et al.*, 1994). Kit 1 and kit 4 presented the highest ratio between free $\beta$-hCG and t-hCG, respectively, demonstrating a higher affinity for the free $\beta$-hCG fraction. This is important when evaluating different t-hCG assays because the free $\beta$-subunit and the free $\beta$/t-hCG ratio varied depending on the stage of tumour progression and higher values are associated with malignant and invasive forms of choriocarcinomas (Fan *et al.*, 1987). As shown in Figure 1 and in Table I, the assay measuring both fractions, intact plus nicked hCG, performed better than the assay measuring intact fraction alone. Recognition of nicked hCG, however, does not seem to be crucial in the use of hCG as a tumour marker in bladder carcinomas, as it was not in testicular cancer (Hoerman *et al.*, 1994).

When chemotherapy was efficient the four kits of t-hCG studied proved to be as useful as free $\beta$-hCG assay; however, in more aggressive forms of bladder carcinoma the assays with the best performance were the free $\beta$-hCG and the t-hCG assays with high free fraction affinity. There is accumulating evidence indicating that the isolated production of free $\beta$-hCG may be associated with aggressive trophoblastic and non-trophoblastic malignancies (Cole *et al.*, 1993; Marcillac *et al.*, 1992; Rinker *et al.*, 1989; Saller *et al.*, 1990).

Like others, (Iles *et al.*, 1989; McLoughlin *et al.*, 1991; Smith *et al.*, 1994), we found no correlation between the hCG concentration and the tumour grade. Moreover, there seems to be no correlation between the hCG concentration and tumour DNA-ploidy and S-phase fraction according to other studies (Fossa *et al.*, 1993).

In conclusion, for the routine use of serum chorionic gonadotropin as a tumour marker in patients with bladder carcinoma, care must be taken in choosing hCG kits as the i-hCG and i-hCG plus hCGn concentration does not reflect the disease and does not detect exclusive secretion of free $\beta$ fraction. On the other hand, t-hCG assays with high affinity for free $\beta$ fraction and the free $\beta$-hCG assay seem to be the most reliable and best choice in the management of aggressive forms of bladder carcinoma.

### Abbreviations hCG, human chorionic gonadotrophin; i-hCG, intact or non-nicked molecule of human chorionic gonadotropin; hCGn, nicked molecule of human chorionic gonadotrophin; free $\beta$-hCG, free beta subunit of human chorionic gonadotrophin; t-hCG, total beta human chorionic gonadotropin (intact molecule plus nicked molecule plus free $\beta$-subunit).

### References

ALFTHAN H, HAGLUND G, ROBERTS P AND STENMAN UH. (1992). Elevation of free $\beta$ subunit of human choriogonadotropin and core $\beta$ fragment of human choriogonadotropin in serum and urine of patients with malignant pancreatic and biliary disease. *Cancer Res.*, **52**, 4628–4633.

COLE LA AND KARDANA A. (1992). Discordant results in human chorionic gonadotropin assays. *Clin. Chem.*, **38**, 263–270

COLE LA, SEIFER DB, KARDANA A AND BRAUNSTEIN GD. (1993). Selecting human chorionic gonadotropin immunoassays: consideration of cross-reacting molecules in first-trimester pregnancy serum and urine. *Am. J. Obstet. Gynecol.*, **168**, 1580–1586.

COLE LA, KOHORN EI AND KIM GS. (1994). Detecting and monitoring trophoblastic disease: new perspective in measuring human chorionic gonadotropin levels. *J. Reprod. Med.*, **39**, 193–200.

DEXEUS F, LOGOTHETIS C, HOSSAN E AND SAMUELS ML. (1986). Carcinoembryonic antigen and betaHCG as serum markers for advanced urothelial malignancies. *J. Urol.*, **136**, 403–407.

FAN C, GOTO S, FURUHASHI Y AND TOMODA Y. (1987). Radioimmunoassay of the serum free β-subunit of human chorionic gonadotropin in trophoblastic disease. *J. Clin. Endocrinol. Methods*, **64**, 313–318.

FOSSA SD, BERNER AA, JACOBSEN AB, WAEHRE H, KVARSTEIN B, URNEST OGREID P, BJERKLUND TE, SILDE J, NESLAND JM AND PETTERSEN EO. (1993). Clinical significance of DNA ploidy and S-phase fraction and their relation to p53 protein, c-*erb*B-2 protein and HCG in operable muscle-invasive bladder cancer. *Br. J. Cancer*, **68**, 572–578.

HATTORI M, YOSHIMOTO Y, MATSUKURA S AND FUJITA T. (1980). Qualitative and quantitative analyses of human chorionic gonadotropin and its subunits produced by malignant tumours. *Cancer*, **46**, 355–361.

HOERMANN R, GERBES AL, SPOETTL G, JÜNGST D AND MANN K. (1992). Immunoreactive human chorionic gonadotropin and its free β subunit in serum and ascites of patients with malignant tumors. *Cancer Res.*, **52**, 1520–1524.

HOERMANN R, BERGER P, SPOETTL G, GILLESBERGER F, KARDANA A, COLE LA AND MANN K. (1994). Immunological recognition and clinical significance of nicked human chorionic gonadotropin in testicular cancer. *Clin. Chem.*, **40**, 2306–2312.

ILES RK AND CHARD T. (1991). Human chorionic gonadotropin expression by bladder cancers: biology and clinical potential. *J. Urol.*, **145**, 453–458.

ILES RK, JENKINS BJ, OLIVER RT, BLANDY JP AND CHARD T. (1989). Beta human chorionic gonadotropin in serum and urine. A marker for metastatic urothelial cancer. *Br. J. Urol.*, **64**, 241–244.

MCLOUGHLIN J, PEPERA T, BRIDGER J, WILLIAMS, G. (1991). Serum and urinary levels of beta HCG in patients with transitional cell carcinomas. (1991). *Br. J. Cancer*, **63**, 822–824.

MADERSBACHER S, KLIEBER R, MANN K, MARTH C, TABARELLI M, WICK G AND BERGER P. (1992). Free α-subunit, free β-subunit of human chorionic gonadotropin (hCG), and intact hCG in sera of healthy individuals and testicular cancer patients. *Clin. Chem.*, **38**, 370–376.

MARCILLAC I, TROALEN F, BIDART JM, GUILLANI P, RIBRAY V AND ESCUDIER B. (1992). Free human chorionic gonadotropin β subunit in gonadal and nongonadal neoplasms. *Cancer Res.*, **52**, 3901–3907.

OLIVER RT, STEPHENSON C, COLLINO CE AND PARKINSON MC. (1988). Clinicopathological significance of immunoreactive beta-hCG production by bladder cancer. *Mol. Biother.*, **1**, 43–45.

PASSING H AND BABLOK W. (1983). A new biometrical procedure for testing the equality of measurements for two different analytical methods. Application of linear regression procedures for method comparison studies in clinical chemistry. Part I. *J. Clin. Chem. Clin. Biochem.*, **21**, 709–720.

RINKER AD AND TIETZ NW. (1989). β-hCG *vs* intact hCG assays in the detection of trophoblastic disease. *Clin. Chem.*, **35**, 1799–1800.

SALLER B, CLARA R, SPOETTL G, SIDDLE K AND MANN K. (1990). Testicular cancer secretes intact human choriogonadotropin (hCG) and its free β-subunit: evidence that hCG (+hCG β) assays are the most reliable in diagnosis and follow-up. *Clin. Chem.*, **36**, 234–239.

SMITH DJ, EVANS HJ, NEWMAN J AND CHAPPLE CR. (1994). Ectopic human chorionic gonadotrophin (HCG) production: is the detection by serum analysis of HCG of clinical relevance in transitional cell carcinoma of the bladder? *Br. J. Urol.*, **73**, 409–412.